# Deciphering clinical significance of BCL11A isoforms and protein expression roles in triple-negative breast cancer subtype

**DOI:** 10.1007/s00432-022-04301-w

**Published:** 2022-08-28

**Authors:** Andrea Angius, Giovanna Pira, Paolo Cossu-Rocca, Giovanni Sotgiu, Laura Saderi, Maria Rosaria Muroni, Patrizia Virdis, Daniela Piras, Rallo Vincenzo, Ciriaco Carru, Donatella Coradduzza, Maria Gabriela Uras, Pierina Cottu, Alessandro Fancellu, Sandra Orrù, Paolo Uva, Maria Rosaria De Miglio

**Affiliations:** 1 Institute of Genetic and Biomedical Research (IRGB), CNR, Cittadella Universitaria Di Cagliari, 09042 Monserrato, CA Italy; 2 Department of Biomedical Sciences, University of Sassari, 07100 Sassari, Italy; 3 Department of Medicine, Surgery and Pharmacy, University of Sassari, 07100 Sassari, Italy; 4 Department of Diagnostic Services, “Giovanni Paolo II” Hospital, ASSL Olbia-ATS Sardegna, 07026 Olbia, Italy; 5 Struttura Complessa Epidemiologia e Registro Tumori Nord Sardegna, ATS Sardegna, 07100 Sassari, Italy; 6 Department of Pathology, “A. Businco” Oncologic Hospital, ARNAS Brotzu, 09121 Cagliari, Italy; 7 IRCCS G. Gaslini, 16147 Genoa, Italy

**Keywords:** Triple negative breast cancer, BCL11A expression, BCL11A mutations, Survival analysis, BCL11A isoforms

## Abstract

**Purpose:**

Triple negative breast cancer (TNBC) is an aggressive clinical tumor, accounting for about 25% of breast cancer (BC) related deaths. Chemotherapy is the only therapeutic option to treat TNBC, hence a detailed understanding of the biology and its categorization is required. To investigate the clinical relevance of BCL11A in TNBC subtype, we focused on gene and protein expression and its mutational status in a large cohort of this molecular subtype.

**Methods:**

Gene expression profiling of BCL11A and its isoforms (BCL11A-XL, BCL11A-L and BCL11A-S) has been determined in Luminal A, Luminal B, HER2-enriched and TNBC subtypes. BCL11A protein expression has been analyzed by immunohistochemistry (IHC) and its mutational status by Sanger sequencing.

**Results:**

In our study, BCL11A was significantly overexpressed in TNBC both at transcriptional and translational levels compared to other BC molecular subtypes. A total of 404 TNBCs were selected and examined showing a high prevalence of BCL11A-XL (37.3%) and BCL11A-L (31.4%) isoform expression in TNBC, associated with a 26% of BCL11A protein expression levels. BCL11A protein expression predicts scarce LIV (HR = 0.52; 95% CI, 0.29–0.92, *P* = 0.03) and AR downregulation (HR = 0.37; 95% CI, 0.16–0.88; *P* = 0.02), as well as a higher proliferative index in TNBC cells. BCL11A-L expression is associated with more aggressive TNBC histological types, such as medullary and metaplastic carcinoma.

**Conclusion:**

Our finding showed that BCL11A protein expression acts as an unfavorable prognostic factor in TNBC patients, especially in non luminal TNBCs subgroups. These results may yield a better treatment strategy by providing a new parameter for TNBC classification.

**Supplementary Information:**

The online version contains supplementary material available at 10.1007/s00432-022-04301-w.

## Introduction

Triple negative breast cancer, which accounts for 10–20% of all invasive breast cancer (BC) subtypes, is characterized by the lack of immunohistochemical expression of estrogen receptor (ER), progesterone receptor (PR), and HER2 and/or HER2 gene amplification. TNBC is most prevalent in women aged < 50 years and shows aggressive clinical behavior (i.e., high histological grade, significantly high metastatic rate and it is responsible for about 25% of BC related deaths) (Angius et al. 2020). Its heterogeneity can be associated with different clinical outcomes. A recent study evaluated the outcome of TNBC patients highlighting that an accurate and reliable histopathologic definition of TNBC subtypes has a significant clinical utility and is an effective tool during the therapeutic decision making process (Sanges et al. 2020). Using gene expression profiling, the molecular signature of TNBC divided the molecular subclassification into four groups: basal-like 1 and 2, mesenchymal, and luminal androgen receptor (LAR) (Lehmann et al. 2016). Gene expression profiling, morphological and immunohistochemical analysis of TNBC represent prognostic and therapeutic tools to customize therapy and improve patient outcomes.

TNBC molecular biomarkers could predict the prognosis (Cagney et al. 2018). We demonstrated that modification of miR-135b might improve the outcome of TNBCs with basal-like features (Uva et al. 2018). The subclassification of patients in our TNBC cohort, based on the high proportion of genetic alterations involving PI3K/AKT pathways, provides evidence that specific genomic abnormalities can select patients who can benefit from targeted therapies (Cossu-Rocca et al. 2015b).

BCL11A was initially detected due to an aberrant chromosomal translocation t(2;14)(p13;q32.3) in human B-cell non-Hodgkin’s lymphomas (Nakamura et al. 2000). BCL11A gene is located on human chromosome 2p13 and is ~ 102 kb in length. BCL11A codes for a protein with an uncommon C2HC zinc finger at the N-terminus and six Krüppel-like C2H2 zinc fingers near the C terminus. Three main mRNA variants were found: BCL11A-XL, BCL11A-L and BCL11A-S, each contains differing numbers of C-terminal C2H2 finger motifs. All 3 isoforms contained the first 3 exons, and only the longest isoform expresses sequences from exons one to four (Satterwhite et al. 2001a). BCL11A-XL protein isoform was expressed in brain and hematopoietic tissues (Liu et al. 2006). Also BCL11A-XL expressed in a range of tumor-derived cell lines (Pulford et al. 2006). Functional studies demonstrated that BCL11A-XL was a transcriptional repressor working in association with itself, other BCL11A isoforms, and with BCL6 gene. So BCL11A-XL might play an essential role in tumor development (Liu et al. 2006; Pulford et al. 2006). High level expression of BCL11A-S was observed in human Hodgkin’s lymphoma cell line [8]. BCL11A-L isoform was expressed preferentially in derived *B*-cell malignant cell lines (Satterwhite et al. 2001a).

Growing evidence demonstrated that BCL11A also plays an essential role in the pathogenesis of solid tumors, including prostate cancer, lung cancer, laryngeal squamous cell carcinoma and acute leukemia (Kapatai and Murray 2007; Chetaille et al. 2009; Boelens et al. 2009; Agueli et al. 2010; Jin et al. 2013; Podgornik et al. 2014). Khaled et al. determined that BCL11A acts as an oncogene in TNBC, and its overexpression is key for tumor formation and invasion. BCL11A supports the development of normal and malignant mammary epithelial stem/progenitor populations (Khaled et al. 2015). Furthermore, its silencing re duces tumor initiating cells population in TNBC xenograft model (Zhu et al. 2019). In the mouse mammary gland, BCL11A is part of a specific subsets of embryonic mammary genes, silenced in adult epithelia and reactivated in mouse and human basal-like breast cancer (Zvelebil et al. 2013). The aim of the present study was to assess the clinical role of BCL11A in the molecular TNBC subtype.

## Methods

A retrospective cohort of BC patients diagnosed between 2000 and 2015 was selected. Samples were obtained from the archives of the Department of Histopathology of the Oncology Hospital of Cagliari, Italy. Inclusion criteria were complete review of surgical specimens and medical records and availability of formalin-fixed, paraffin-embedded (FFPE) tumor blocks from surgical specimens. Three experienced pathologists independently reviewed all cases. Histologic subtyping was performed according to current WHO classification (Rakha et al. 2019). Three µm thick tissue sections of FFPE specimens were cut for hematoxylin and eosin staining, IHC, in situ hybridization (SISH) and genetic analysis. The study protocol was approved by the Azienda Sanitaria Locale Sassari Bioethics Committee (n. 1140/L, 05/21/2013); and followed the Italian law on guidelines for the implementation of retrospective observational studies (G.U. n. 76, 31 March 2008). Only coded data were collected to protect patient confidentiality.

### Immunohistochemistry

ER, PR, HER2 and Ki-67 immunohistochemical expression and/or HER2 gene amplification, as defined by silver enhanced SISH, established the surrogate intrinsic subtypes of BC, based on the St. Gallen Consensus 2013 (Goldhirsch et al. 2011). AR Clone SP107 (Cell-MarqueTM, Rocklin, CA, USA) was used to determine AR expression. IHC and SISH analysis were performed as previously described (Orrù et al. 2022). BCL11A clone 14B 5 (dilution 1:100, ab19487, AbCam, Cambridge, USA) was used to determine BCL11A expression. The ab19487 antibody, whose epitope is in core of amino acids 172–434, can identify the BCL11A-XL and BCL11A-L isoforms. BCL11A immunostaining was performed using the Ventana Benchmark XT staining system with an Optiview DAB detection kit. IHC analysis was performed on 87 BC and 12 normal breast tissue (NBT) FFPE block samples. Also, 343 TNBC tissue microarrays (TMAs) were used.

### Evaluation of immunohistochemical staining

ER and PR expression were positive if at least 1% immunostained tumor nuclei were detected in the sample, according to the American Society of Clinical Oncology/College of American Pathologists (ASCO/CAP) recommendations for immunohistochemical testing of hormone receptors in BC (Hammond et al. 2010), whose criteria have recently been adopted by WHO classification (Rakha et al. 2019). The Ki67 cut-offs < 14, 15–35% and > 35% were based on results previously obtained (Urru et al. 2018); AR expression was considered positive if at least 10% immunostained tumor nuclei were detected in the sample (Park et al. 2010). All IHC expressions were categorized using a semi-quantitative method.

Based on IHC approach the following BC surrogate intrinsic subtypes were found: nine Luminal A [ER and PR expression positive, with PR cut point of ≥ 20%, HER2 negative and Ki-67 low (< 14%)]; nine Luminal B [ER expression positive, PR expression negative or low, HER2 expression negative and Ki-67 high (> 14%), or ER expression positive, HER2 protein positive or HER2 gene amplified, any PR and any Ki-67]; eight HER2-enriched [ER and PR expression negative, HER2 protein positive or HER2 gene amplified]; sixty-one TNBC [ER, PR and HER2 expression negative or HER2 gene not amplified]. The ordinal Allred scoring system was used to assess BCL11A immunostaining quantity in tumor cells, based on intensity (0, negative; 1 + , weak; 2 + , moderate; 3 + , strong) and percentage of stained cells (0 = 0%, 1 =  < 1%, 2 = 1–10%, 3 = 11–33%, 4 = 34–66% and 5 =  > 66%); the combination of intensity + percentage gives an Allred score between 0 and 8. Tumor with Allred score > 2 was defined as positive for BCL11A expression (Khaled et al. 2015).

### Acid nucleic extraction

Genomic DNA was obtained from neoplastic tissue, and total RNA was obtained from neoplastic and non-neoplastic specimens. Nucleic acids were extracted using the QIAmp DNA Mini Kit and miRNeasy Mini Kit (Qiagen, Hilden, Germany). The quantity and the quality of nucleic acids were assessed using Nanodrop ND1000 (Euro-Clone, Milan, Italy). The RNA quantity was evaluated by Qubit^®^ RNA BR Assay Kit (ThermoFisher Scientific, Waltham, USA). The RNA integrity was assessed by the RNA Integrity Number (RIN) using the Agilent RNA 6000 Nano Kit on the BioAnalyzer 2100 (Agilent, Santa Clara, USA).

### Quantitative real time PCR

Gene expression profiles of BCL11A were analyzed in all BC molecular intrinsic subtypes. Two µg of total RNA were reverse transcribed to cDNA using the High-Capacity cDNA Reverse Transcription Kit (Applied Biosystem, Foster City, CA, USA). BCL11A encodes three mRNA variants and each isoform of BCL11A has specific expression patterns. Primers for BCL11A (Hs01076078_m1, 60 bp), the isoforms BCL11A-S (Hs01093198_m1), BCL11A-L (Hs01093199-m1), BCL11A-XL (Hs00250581_s1) and 18S rRNA (Hs99999901_S1, 187 bp) human genes were chosen using Assays-on-Demand™-Products (Applied Biosystems). Neoplastic and non-neoplastic tissues were analyzed by quantitative real time PCR (qRT-PCR) using the ABI 7900HT Sequence Detection System (Applied Biosystems) (Cossu-Rocca et al. 2015a). The relative mRNA expression level was analyzed according to the Applied Biosystem User Bulletin N°2. The calculation 2-ΔΔCt (Fold Change, FC) was chosen to represent the level of expression, with a FC > 2 being considered as overexpression.

### Mutation analysis

BCL11A gene mutation analysis was performed on exon 4 encoding five of the six Kruppel-like zinc-finger domains (C2H2) of the BCL11A-XL protein, where several most common missense mutations were identified in patients affected by autism, intelligence disabilities (Cai et al. 2017), and ovarian cancer (Er et al. 2016): the exon 4 contains almost all the BCL11A single nucleotide polymorphisms. Amplification of the exon 4 and Sanger sequencing analysis were performed in all BC molecular subtypes analyzed for gene expression profile, using the following sequence primers: BCL11A_ex4_F2:5ʹ-ACCGCATAGACGATGGCAC-3ʹ and BCL11A_ex4_R2:5ʹ-CCCCGAGATCCCTCCGT-3ʹ (De Miglio et al. 2010).

### Statistical analysis

An ad hoc electronic form was created to collect qualitative and quantitative variables. Qualitative data were summarized with absolute and relative (percentages) frequencies. Chi-squared or Fisher exact tests were used to detect any statistical differences in the comparison of qualitative variables between down and up regulation of BCL11A gene or low and high protein expression. Logistic regression analysis was performed to assess the relationship between BCL11A upregulation or high protein expression and clinicopathological TNBC characteristics. Survival rate differences between down and upregulation or low and high protein expression were detected with Kaplan–Meier analysis. P-value less than 0.05 was considered statistically significant. Stata 17 (StataCorp, TX) statistical software was used for every statistical computation.

## Results

### BCL11A expression in molecular intrinsic subtypes of breast cancer

**Fig. 1:**
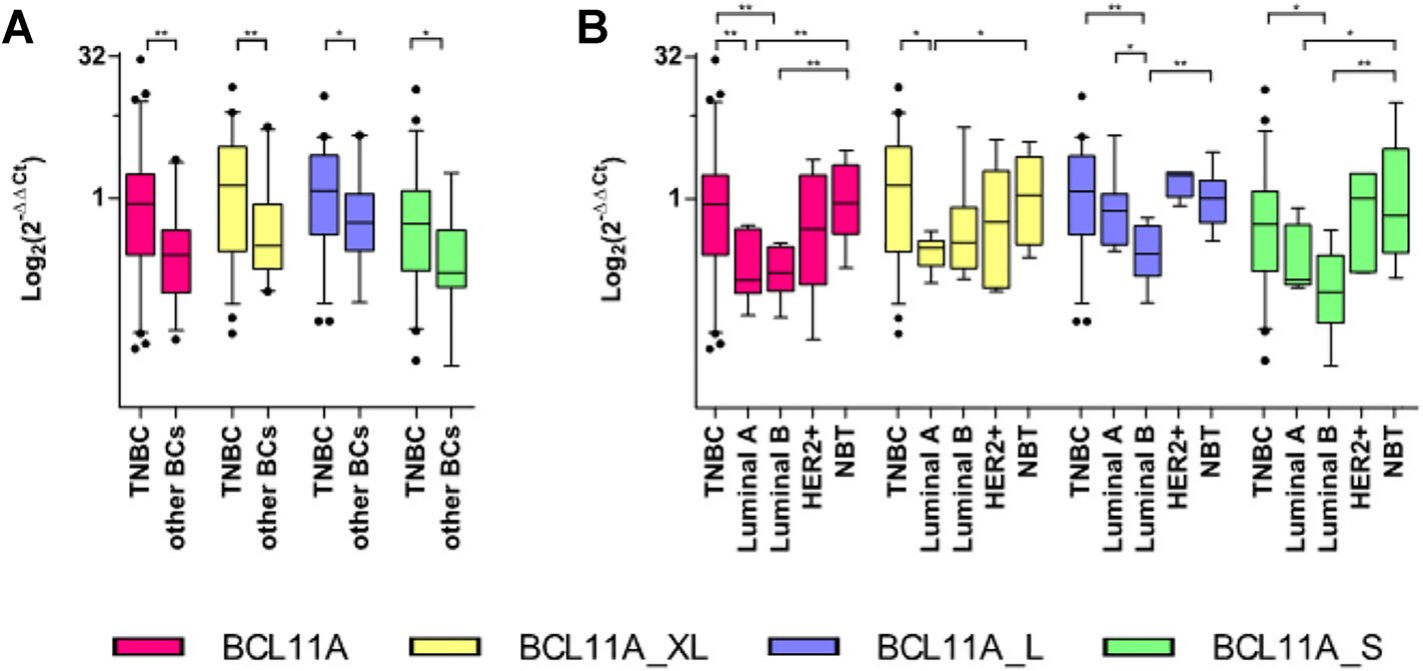
Expression of BCL11A and its mRNA isoforms in molecular intrinsic subtypes of breast cancer. **A** Significant BCL11A expression in TNBC compared to other molecular intrinsic subtypes of breast cancer. **B** BCL11A mRNA expression across the molecular intrinsic subtypes of breast cancer. Mann–Whitney test was used. *p-value < 0.05; **p-value < 0.01

Eighty-seven primary BC, comprising all molecular subtypes, were analyzed by gene expression profiling by qRT-PCR. The overall high expression of BCL11A and each of its transcripts (BCL11A-XL, BCL11A-L and BCL11A-S) significantly correlated with TNBC pathology (*P* < 0.05) (Fig. 1A).

We found a significant BCL11A overexpression in TNBC compared to Luminal A (P: 0.004) and B (P: 0.002) while a significant BCL11A downregulation was present in Luminal A and B compared to NBT (P: 0.002 and *P* < 0.001, respectively). No significant differences were shown between HER2-enriched and other molecular intrinsic subtypes and NBT. BCL11A-XL was overexpressed in TNBC vs Luminal A and B (P: 0.012 and P: 0.040, respectively), whereas BCL11A-L and BCL11A-S were overexpressed in TNBC vs Luminal B (P: 0.003 and P: 0.011, respectively) (Fig. 1B).

**Fig. 2:**
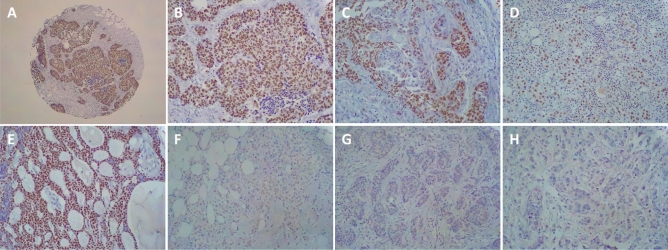
BCL11A immunohistochemical expression in molecular intrinsic breast cancer subtypes. **A** Immunohistochemistry for BCL11A displaying diffuse and intense immunoreactivity in TNBC (IBC-NST, original magnification 40 ×); **B** Immunohistochemistry for BCL11A displaying diffuse and intense immunoreactivity in TNBC (IBC-NST, original magnification 200 ×); **C** Immunohistochemistry for BCL11A displaying diffuse and intense immunoreactivity in TNBC (Medullary-type carcinoma, original magnification 200 ×); **D** Immunohistochemistry for BCL11A displaying diffuse and intense immunoreactivity in TNBC (Metaplastic carcinoma, original magnification 40 ×); **E** Immunohistochemistry for BCL11A displaying diffuse and intense immunoreactivity in TNBC (adenoid cystic carcinoma, original magnification 40 ×); **F** Negative immunohistochemistry for BCL11A in Luminal A breast cancer (IBC-NST, original magnification 200 ×); **G** Negative immunohistochemistry for BCL11A in Luminal B breast cancer (IBC-NST, original magnification 200 ×); **H** Negative immunohistochemistry for BCL11A in HER2-enriched breast cancer (Invasive lobular carcinoma, original magnification 200 ×)

Focusing on BCL11A protein expression profile we performed IHC on the 87 primary BC molecular subtypes used for gene expression profile. A BCL11A protein overexpression was found in 16 out of 61 (26.2%) TNBCs, in 4 out of 12 (33.3%) NBT, whereas no protein expression was detected in Luminal A (nine cases), Luminal B (nine cases) and HER2-enriched (eight cases) tumors. The tumors immunostained positively showed high mRNA levels compared with those with negative immunostaining. Immunohistochemistry analysis of BCL11A in an independent validation cohort of 343 TNBC samples, confirmed that BCL11A protein expression agreed with the first cohort examined: 79 BCL11A-overexpressing TNBCs out of 343 (23.0%). Figure 2 showed a representative BCL11A protein expression of BC molecular intrinsic subtypes.

### BCL11A expression profile and association with TNBC clinic-pathological data

Table 1 showed the clinic-pathological features of the 61 TNBC patients included in the expression profile analysis. The median (interval quartile range, IQR) age at diagnosis was 57 (31–84) years, with 39 (63.9%) older than 50 years. Forty (65.6%) tumors were ductal, 9 (14.8%) medullary, 4 (6.6%) metaplastic. Tumor staging was pT1 in 24 (42.9%) cases, pT2 in 26 (46.4%), pT3 in 3 cases (5.4%), pT4 in 3 cases (5.4%). Lymph node status was divided into 31 pN0 (53.5%), 16 pN1 (27.6%), 6 pN2 (10.3%) and 5 pN3 (8.6%).

**Table 1:** Clinico-pathological features of 61 patients with triple negative breast cancer

Variables		*N* (%)
Median (IQR) age, years		57 (31–84)
Age, *n* (%)	≤ 50 years	22 (36.1)
	> 50 years	39 (63.9)
Menstrual status at referred, *n* (%)	Yes, physiological	13 (46.4)
	Yes, post-surgical	4 (46.4)
	No	11 (39.3)
Site, *n* (%)	Right	31 (53.5)
	Left	26 (44.8)
	Bilateral	1 (1.7)
Histologic subtype, *n* (%)	Invasive ductal carcinoma (NST)	40 (65.6)
	Medullary carcinoma	9 (14.8)
	Metaplastic carcinoma	4 (6.6)
	Other	8 (13.1)
Tumor size, *n* (%)	≤ 20 mm	34 (55.7)
	> 20 mm	27 (44.3)
Pathologic tumor classification, *n* (%)	pT1	24 (42.9)
	pT2	26 (46.4)
	pT3	3 (5.4)
	pT4	3 (5.4)
Regional lymph nodes involvement, *n* (%)	pN0	31 (53.5)
	pN1	16 (27.6)
	pN2	6 (10.3)
	pN3	5 (8.6)
Histologic grade, *n* (%)	G1	3 (4.9)
	G2	8 (13.1)
	G3	50 (82.0)
Tumor stage, *n* (%)	1	13 (24.1)
	2	29 (53.7)
	3	12 (22.2)
Necrosis, *n* (%)	Present	20 (35.1)
	Absent	
Tumor infiltrating lymphocytes, *n* (%)	Present	27 (52.9)
	Absent	24 (40.1)
Lymph vascular invasion, *n* (%)	Present	13 (25.5)
	Absent	38 (74.5)
Ki67, *n* (%)	≤ 20%	12 (19.7)
	> 20%	49 (80.3)
AR, *n* (%)	< 10%	38 (69.1)
	≥ 10%	17 (30.9)
Death, *n* (%)		8 (13.1)
Gene expression BCL11A	Down	47 (77.1)
	Up	14 (23.0)
Gene expression BCL11A_XL	Down	37 (62.7)
	Up	22 (37.3)
Gene expression BCL11A_L	Down	35 (68.6)
	Up	16 (31.4)
Gene expression BCL11A_S	Down	42 (87.5)
	Up	6 (12.5)
Protein expression BCL11A	Low: 0–1	45 (73.8)
	High: 2–8	16 (26.2)

*IQR* interquartile range, *N* number

Moreover, 24.1% of tumors were stage I, 53.7% stage II, and 22.2% stage III; 4.9% of TNBCs were G1, 13.1% G2, and 82.0% G3. Ki-67 expression was > 20% in 80.3% of TNBCs. Necrosis was present in 35.1%. Tumor infiltrating lymphocyte (TIL) and lymphovascular invasion (LVI) were detected in 52.9 and 25.5%, respectively. AR expression was found in 30.9% cases. A total of 8 patients out of 61 (13.1%) died. The clinicopathological data of the validation cohort is reported in Table S1. TNBCs with BCL11A and BCL11A-L mRNA overexpression were more frequently associated with AR expression < 10% (*P*: 0.05). BCL11A-L mRNA overexpression was associated with some histological types such as medullary and metaplastic carcinomas (P: 0.04) (Table 2).

**Table 2:** Clinico-pathological data of 61 patients with triple negative breast cancer based on BCL11A gene expression

	BCL11A			BCL11A_XL			BCL11A_L			BCL11A_S		
	Down	Up	*p*-value	Down	Up	*p*-value	Down	Up	*p*-value	Down	Up	*p*-value
Age at diagnosis (years)												
≤ 50	15 (31.9)	7 (50.0)	0.22	12 (32.4)	9 (40.9)	0.51	10 (28.6)	8 (50.0)	0.14	14 (33.3)	4 (66.7)	0.18
> 50	32 (68.1)	7 (50.0)		25 (67.6)	13 (59.1)		25 (71.4)	8 (50.0)		28 (66.7)	2 (33.3)	
Menstrual Status												
Yes, physiological	11 (50.0)	2 (33.3)	0.83	9 (47.4)	3 (37.5)	0.85	8 (44.4)	2 (40.0)	0.98	10 (47.6)	0 (0.0)	0.31
Yes, post-surgical	3 (13.6)	1 (16.7)		3 (15.8)	1 (12.5)		3 (16.7)	1 (20.0)		4 (19.1)	0 (0.0)	
No	8 (36.4)	3 (50.0)		7 (36.8)	4 (50.0)		7 (38.9)	2 (40.0)		7 (33.3)	2 (100.0)	
Site												
Right	21 (47.7)	10 (71.4)	0.41	18 (52.9)	12 (54.6)	1.00	16 (48.5)	9 (56.3)	0.72	17 (42.5)	5 (83.3)	0.21
Left	22 (50.0)	4 (28.6)		15 (44.1)	10 (45.5)		16 (48.5)	9 (56.3)		22 (55.0)	1 (16.7)	
Bilateral	1 (2.3)	0 (0.0)		1 (2.9)	0 (0.0)		1 (3.0)	0 (0.0)		1 (2.5)	0 (0.0)	
Histological subtype												
Invasive ductal carcinoma (NST)	32 (68.1)	8 (57.1)	0.80	27 (73.0)	13 (59.1)	0.36	27 (77.1)	7 (43.8)	0.04	29 (69.1)	2 (33.3)	0.17
Medullary carcinoma	6 (12.8)	3 (21.4)		3 (8.1)	5 (22.7)		3 (8.6)	4 (25.0)		5 (11.9)	2 (33.3)	
Metaplastic carcinoma	3 (6.4)	1 (7.1)		2 (5.4)	2 (9.1)		1 (2.9)	3 (18.8)		3 (7.1)	1 (16.7)	
Other	6 (12.8)	2 (14.3)		5 (13.5)	2 (9.1)		4 (11.4)	2 (12.5)		5 (11.9)	1 (16.7)	
Tumor size (cm)												
≤ 20 mm	26 (55.3)	8 (57.1)	0.90	19 (51.4)	13 (59.1)	0.56	18 (51.4)	10 (62.5)	0.46	24 (57.19	3 (50.0)	1.00
> 20 mm	21 (44.7)	6 (42.9)		18 (48.7)	9 (40.9)		17 (48.6)	6 (37.5)		18 (42.9)	3 (50.0)	
Histologic grade												
G1	1 (2.1)	2 (14.3)	0.20	1 (2.7)	2 (9.1)	0.38	1 (2.9)	2 (12.5)	0.28	2 (4.8)	1 (16.7)	0.25
G2	7 (14.9)	1 (7.1)		4 (10.8)	4 (18.2)		5 (14.3)	3 (18.8)		8 (19.1)	0 (0.0)	
G3	39 (83.0)	11 (78.6)		32 (86.5)	16 (72.7)		29 (82.9)	11 (68.8)		32 (76.2)	5 (83.3)	
Pathologic tumor classification												
pT1	17 (38.6)	7 (58.3)	0.65	11 (32.4)	11 (55.0)	0.20	11 (35.5)	8 (53.3)	0.39	16 (42.1)	2 (40.0)	1.00
pT2	21 (47.7)	5 (41.7)		17 (50.0)	9 (45.0)		14 (45.2)	7 (46.7)		17 (44.7)	3 (60.0)	
pT3	3 (6.8)	0 (0.0)		3 (8.8)	0 (0.0)		3 (9.7)	0 (0.0)		3 (7.9)	0 (0.0)	
pT4	3 (6.8)	0 (0.0)		3 (8.8)	0 (0.0)		3 (9.7)	0 (0.0)		2 (5.3)	0 (0.0)	
Regional lymph nodes involvement												
pN0	23 (52.3)	8 (57.1)	0.68	16 (45.7)	13 (61.9)	0.33	14 (42.4)	8 (53.3)	0.44	18 (46.2)	3 (50.0)	0.84
pN1	11 (25.0)	5 (35.7)		10 (28.6)	6 (28.6)		10 (30.3)	6 (40.0)		12 (30.8)	3 (50.0)	
pN2	5 (11.4)	1 (7.1)		4 (11.4)	2 (9.5)		4 (12.1)	1 (6.7)		5 (12.8)	0 (0.0)	
pN3	5 (11.4)	0 (0.0)		5 (14.3)	0 (0.0)		5 (15.2)	0 (0.0)		4 (10.3)	0 (0.0)	
Tumor stage												
I	10 (23.8)	3 (25.0)	0.46	5 (15.2)	6 (31.6)	0.18	6 (20.0)	3 (21.4)	0.18	8 (22.2)	0 (0.0)	0.21
II	21 (50.0)	8 (66.7)		18 (54.6)	11 (57.9)		14 (46.7)	10 (71.49		18 (50.0)	5 (100.0)	
III	11 (26.2)	1 (8.3)		10 (30.3)	2 (10.5)		10 (33.3)	1 (7.1)		10 (27.8)	0 (0.0)	
Necrosis												
Present	16 (37.2)	4 (28.6)	0.75	15 (44.1)	5 (22.7)	0.10	12 (36.4)	4 (25.0)	0.53	13 (32.5)	2 (33.3)	1.00
Absent	27 (62.8)	10 (71.4)		19 (55.9)	17 (77.3)		21 (63.6)	12 (75.0)		27 (67.5)	4 (66.7)	
Tumor infiltrating lymphocytes												
Present	20 (52.6)	7 (53.9)	1.00	15 (51.7)	11 (52.4)	1.00	13 (46.4)	8 (53.3)	0.76	16 (44.4)	4 (80.0)	0.18
Absent	18 (47.4)	6 (46.2)		14 (48.3)	10 (47.6)		15 (53.6)	7 (46.7)		20 (55.6)	1 (20.0)	
Lymph vascular invasion												
Present	8 (21.1)	5 (38.5)	0.21	7 (24.1)	6 (28.6)	0.72	7 (25.0)	3 (20.0)	1.00	8 (22.2)	2 (40.0)	0.58
Absent	30 (78.9)	8 (61.5)		22 (75.9)	15 (71.4)		21 (75.0)	12 (80.0)		28 (77.8)	3 (60.0)	
Ki 67												
≤ 20%	11 (23.9)	1 (7.1)	0.26	8 (22.2)	3 (13.6)	0.51	9 (26.5)	1 (6.3)	0.14	9 (22.0)	0 (0.0)	0.58
> 20%	35 (76.1)	13 (92.9)		28 (77.8)	19 (86.4)		25 (73.5)	15 (93.8)		32 (78.1)	6 (100.0)	
Androgen receptor												
< 10%	26 (61.9)	12 (92.3)	0.05	20 (60.6)	18 (85.7)	0.07	18 (56.3)	13 (86.7)	0.05	23 (59.0)	5 (100.0)	0.14
≥ 10%	16 (38.1)	1 (7.7)		13 (39.4)	3 (14.3)		14 (43.8)	2 (13.3)		16 (41.0)	0 (0.0)	
Protein expression BCL11A												
Low: 0–3	41 (87.2)	4 (28.6)	< 0.0001	34 (91.9)	9 (40.9)	< 0.0001	31 (88.6)	7 (43.8)	0.0001	34 (81.0)	2 (33.3)	0.03
High: 4–8	6 (12.8)	10 (71.4)		3 (8.1)	13 (59.1)		4 (11.4)	9 (56.3)		8 (19.1)	4 (66.7)	
Death	7 (14.9)	1 (7.1)	0.67	7 (18.9)	1 (4.6)	0.24	7 (20.0)	1 (6.3)	0.41	7 (16.7)	0 (0.0)	0.57

BCL11A protein expression was associated with ki-67 > 35% (P: 0.004), and with absence of LIV and AR downregulation (P: 0.03 and P: 0.02, respectively) (Table 3).

**Table 3:** Clinico-pathological data of 404 patients with triple negative breast cancer based on BCL11A protein expression

	Protein expression BCL11A		*p*-value
	Low	High	
Age at diagnosis (years)			
≤ 50	109 (35.3)	34 (36.2)	0.87
> 50	200 (64.7)	60 (63.8)	
Site			
Right	119 (44.9)	45 (55.6)	0.11
Left	145 (54.7)	35 (43.2)	
Bilateral	1 (0.4)	1 (1.2)	
Histological subtype			
Invasive ductal carcinoma (NST)	202 (71.4)	68 (76.4)	0.09
Apocrine carcinoma	19 (6.7)	2 (2.3)	
Medullary carcinoma	17 (6.0)	12 (13.5)	
Invasive lobular carcinoma	13 (4.6)	2 (2.3)	
Metaplastic + squamous carcinoma	12 (4.2)	1 (1.1)	
Papillary carcinoma	5 (1.8)	0 (0.0)	
Other	15 (5.3)	4 (4.5)	
Tumor size (cm)			
≤ 20 mm	106 (47.5)	32 (45.1)	0.72
> 20 mm	117 (52.5)	39 (54.9)	
Histologic grade			
G1	4 (1.4)	3 (3.2)	0.13
G2	45 (15.2)	8 (8.6)	
G3	247 (83.5)	82 (88.2)	
Pathologic tumor classification			
pT1	102 (35.4)	36 (39.1)	0.82
pT2	145 (50.4)	45 (48.9)	
pT3	23 (8.0)	5 (5.4)	
pT4	18 (6.3)	6 (6.5)	
Regional lymph nodes involvement			
pN0	156 (54.9)	54 (60.0)	0.16
pN1	70 (24.7)	27 (30.0)	
pN2	32 (11.3)	5 (5.6)	
pN3	26 (9.2)	4 (4.4)	
Tumor stage			
I	67 (26.0)	17 (23.6)	0.21
II	126 (48.8)	44 (61.1)	
III	59 (22.9)	11 (15.3)	
IV	6 (2.3)	0 (0.0)	
Necrosis			
Present	127 (56.7)	43 (55.1)	0.81
Absent	97 (43.3)	35 (44.9)	
Tumor infiltrating lymphocytes			
Present	151 (69.6)	46 (61.3)	0.19
Absent	66 (30.4)	29 (38.7)	
Lymph vascular invasion			
Present	91 (42.7)	20 (27.8)	0.03
Absent	122 (57.3)	52 (72.2)	
Ki67			
≤ 14%	21 (7.0)	3 (3.2)	0.004
15–35%	85 (28.3)	13 (14.0)	
> 35%	194 (64.7)	77 (82.8)	
Androgen receptor			
< 10%	128 (72.7)	50 (87.7)	0.02
≥ 10%	48 (27.3)	7 (12.3)	
Death	105 (34.5)	27 (28.7)	0.30

### BCL11A expression profile and association with TNBC survival

Logistic regression analysis revealed that histological type (HR, 0.2; 95% CI 0.1–0.8; P: 0.02) and AR expression (HR, 0.2; 95% CI 0.0–1.0; P: 0.05) are independent prognostic factors for overall survival (OS) in BCL11A-L transcripts overexpressing TNBCs. High protein expression levels of BCL11A (HR, 17.1; 95% CI 4.0–72.2; *P* < 0.001) are independent prognostic factors for TNBCs overexpressing mRNA BCL11A or its isoforms (Tables 4).

**Table 4:** Univariate analysis for overall survival in 61 patients with triple negative breast cancer expressing BCL11A gene

Variables		BCL11A		BCL11A-XL		BCL11A-L		BCL11A-S	
		OR (95% CI)	*p*-value	OR (95% CI)	*p*-value	OR (95% CI)	*p*-value	OR (95% CI)	*p*-value
Age > 50 years		0.5 (0.1–1.6)	0.22	0.7 (0.2–2.1)	0.51	0.4 (0.1–1.4)	0.14	0.3 (0.0–1.5)	0.13
Right site		2.7 (0.8–10.1)	0.13	1.1 (0.4–3.1)	0.91	1.4 (0.4–4.5)	0.61	6.8 (0.7–63.3)	0.09
Invasive ductal carcinoma (NST)		0.6 (0.2–2.1)	0.45	0.5 (0.2–1.69	0.27	0.2 (0.1–0.8)	0.02	0.2 (0.0–1.4)	0.11
Tumor size > 20 mm		0.9 (0.3–3.1)	0.90	0.7 (0.3–2.1)	0.56	0.6 (0.2–2.1)	0.46	1.3 (0.2–7.4)	0.74
Pathologic tumor classification	pT1	Ref	Ref	Ref	Ref	Ref	Ref	Ref	Ref
	pT2	0.6 (0.2–2.2)	0.41	0.5 (0.2–1.7)	0.28	0.7 (0.2–2.5)	0.57	1.4 (0.2–9.6)	0.72
	pT3	–	–	–	–	–	–	–	–
	pT4	–	–	–	–	–	–	–	
Regional lymph nodes involvement	pN0	Ref	Ref	Ref	Ref	Ref	Ref	Ref	Ref
	pN1	1.3 (0.4–4.9)	0.69	0.7 (0.2–2.6)	0.63	1.1 (0.3–4.0)	0.94	1.5 (0.3–8.7)	0.65
	pN2	0.6 (0.1–5.7)	0.64	0.6 (0.1–3.9)	0.61	0.4 (0.0–4.6)	0.49	–	–
	pN3	–	–	–	–	–	–	–	–
Histologic grade	G1	Ref	Ref	Ref	Ref	Ref	Ref	Ref	Ref
	G2	0.07 (0.003–1.73)	0.11	0.5 (0.03–8.0)	0.62	0.3 (0.0–4.9)	0.40	–	–
	G3	0.14 (0.01–1.70)	0.12	0.3 (0.02–3.0)	0.27	0.2 (0.0–2.3)	0.19	0.3 (0.02–4.12)	0.38
Tumor stage	I	Ref	Ref	Ref	Ref	Ref	Ref	–	–
	II	1.3 (0.3–5.8)	0.76	0.5 (0.1–2.1)	0.35	1.4 (0.3–7.1)	0.66	–	–
	III	0.30 (0.03–3.41)	0.33	0.2 (0.02–0.14)	0.07	0.2 (0.02–2.39)	0.20	–	–
Necrosis		0.7 (0.2–2.5)	0.56	0.4 (0.1–1.2)	0.11	0.6 (0.2–2.2)	0.43	1.0 (0.2–6.4)	0.97
Tumor infiltrating lymphocytes		1.1 (0.3–3.7)	0.94	1.0 (0.3–3.2)	0.96	1.3 (0.4–4.6)	0.67	5.0 (0.5–49.3)	0.17
Lymph vascular invasion		2.3 (0.6–9.2)	0.22	1.3 (0.4–4.5)	0.73	0.8 (0.2–3.5)	0.71	2.3 (0.3–16.5)	0.40
Ki 67 > 20		4.1 (0.5–34.9)	0.20	1.8 (0.4–7.7)	0.42	5.4 (0.6–47.0)	0.13	–	–
AR ≥ 10		0.1 (0.0–1.1)	0.07	0.3 (0.1–1.1)	0.06	0.2 (0.0–1.0)	0.05	–	–
High protein expression BCL11A		17.1 (4.0–72.2)	< 0.0001	16.4 (3.8–70.1)	< 0.0001	10.0 (2.4–41.9)	0.002	8.5 (1.3–54.8)	0.02

LIV (HR, 0.52; 95% CI 0.29–0.92; P: 0.03) and AR (HR, 0.37; 95% CI 0.16–0.88; P: 0.02) are independent prognostic factors for TNBCs showing high BCL11A protein expression levels (Table 5).

**Table 5:** Univariate analysis for overall survival in 404 patients with triple negative breast cancer expressing BCL11A protein

Variables		OR (95% CI)	*p*-value
Age > 50 years		0.96 (0.59–1.56)	0.87
Right site		1.53 (0.93–2.53)	0.09
Invasive ductal carcinoma (NST)		1.30 (0.75–2.26)	0.36
Tumor size > 20 mm		1.10 (0.65–1.89)	0.72
Pathologic tumor classification	pT1	Ref	Ref
	pT2	0.88 (0.53–1.46)	0.62
	pT3	0.62 (0.22–1.74)	0.36
	pT4	0.94 (0.35–5.57)	0.91
Regional lymph nodes involvement	pN0	Ref	Ref
	pN1	1.11 (0.65–1.91)	0.70
	pN2	0.45 (0.17–0.22)	0.12
	pN3	0.44 (0.15–1.33)	0.15
Histologic grade	G1	Ref	Ref
	G2	0.24 (0.4–1.27)	0.09
	G3	0.44 (0.10–2.02)	0.29
Tumor stage	I	Ref	Ref
	II	1.38 (0.73–2.59)	0.32
	III	0.74 (0.32–1.69)	0.47
	IV	-	-
Necrosis		0.94 (0.56–1.58)	0.81
Tumor infiltrating lymphocytes		0.69 (0.40–1.20)	0.19
Lymph vascular invasion		0.52 (0.29–0.92)	0.03
Ki 67 > 20%	≤ 14%	Ref	Ref
	15–35%	1.07 (0.28–4.10)	0.92
	> 35%	2.78 (0.81–9.58)	0.11
AR ≥ 10		0.37 (0.16–0.88)	0.02

**Fig. 3:**
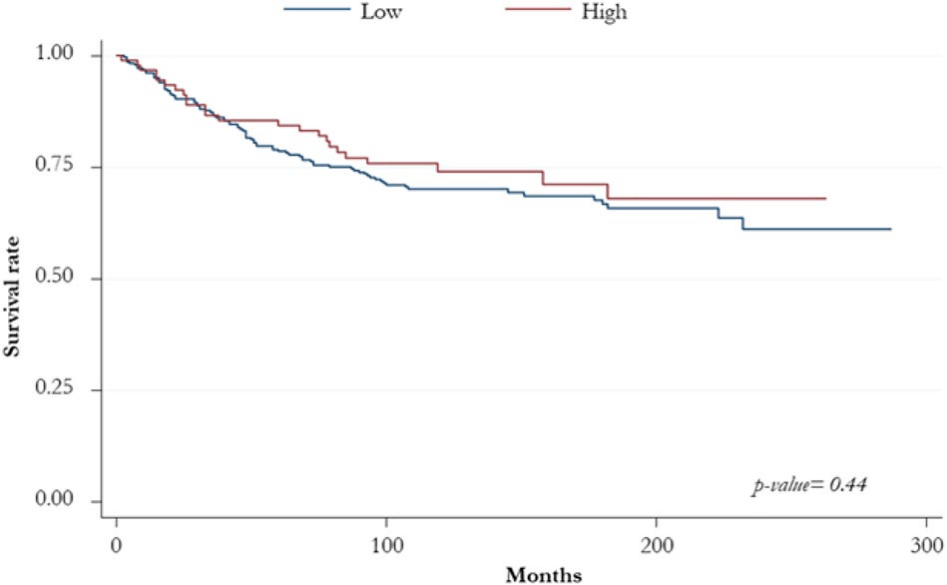
Kaplan–Meier curves for overall survival according to BCL11A protein expression in all TNBC cohort

Kaplan–Meier curve for OS showed no differences among TNBCs with overexpression of BCL11A transcripts and its isoforms in comparison with those downregulated. We observed the same trend for TNBCs with high protein expression levels, analyzing the entire cohort of tumors included in the study (Fig. 3).

### BCL11A mutational analysis in molecular intrinsic subtypes of breast cancer

Sequencing of BCL11A exons 4 did not find any genomic variation in our BC molecular cohort, expect the rs7569946. This synonymous substitution C vs T (Phe699Phe), was detected in all BC molecular subtypes. CC genotype was prevalent in all BC molecular subtypes (60–62.5%). In TNBC subtype, no TT homozygous were present while 40% of them showed CT genotype.

## Discussion

BCL11A is a proto-oncogene which maps on chromosome 2p16. Alternative splicing generates at least three most common BCL11A transcripts, BCL11A-XL, BCL11A-L and BCL11A-S containing differing numbers of C-terminal C2H2 finger motifs, and showing low expression in normal human tissue, except in fetal liver, hematopoietic tissue and brain (Yin et al. 2019). The BCL11A-XL mRNA is the prevalent transcript (Satterwhite et al. 2001b). BCL11A acts as a transcription repressor directly binding to its DNA target sequence, 5ʹ-GGCCGG-3ʹ (Avram et al. 2002) and/or indirectly interacting with and repressing other sequence specific transcription factors, such as COUP-TFs (Avram et al. 2000).

BCL11A is an oncogene of different malignant hematological diseases (Weniger et al. 2006; Nakamura et al. 2000). Recently, the pathogenetic role of BCL11A was also highlighted in solid tumors (e.g., lung, prostate, breast cancer, endometrial carcinoma, laryngeal squamous carcinoma) (Zhang et al. 2015, 2016; Jiang et al. 2013; Khaled et al. 2015; Zhou et al. 2017; Chen et al. 2018; Wang et al. 2020).

In our study, BCL11A was significantly overexpressed in TNBC both at transcriptional and translational levels compared to the other BC molecular subtypes. Gene expression profiling showed that high expression levels of BCL11A and its isoforms (BCL11A-XL, BCL11A-L and BCL11A-S) significantly correlated with TNBC pathology. Additionally, tumors positively immunostained showed high BCL11A mRNA levels compared with those with negative immunostaining. Our results confirmed recent data correlating BCL11A overexpression and TNBC subtype (Khaled et al. 2015). We found BCL11A protein expression in 26% of TNBCs in our cohort, likewise to the 29.6% reported by Chen et al. (Chen et al. 2018), in contrast with Khaled et al. (67% of BCL11A expression in TNBC with basal-like features) (Khaled et al. 2015) and Wang et al. (100% of BCL11A expression in TNBC using a different score to define BCL11A overexpression) (Wang et al. 2020). The lower percentage of BCL11A protein expression detected in our cohort could depend on several factors: the definition of BCL11A expression by several operators, the cut-off values used, or the analysis performed on all TNBCs despite classification into molecular sub-classes.

Regarding the prognostic significance, we showed that BCL11A protein expression acts as an unfavorable prognostic factor in TNBC patients. Metaplastic and medullary histotypes, absence of LIV and AR downregulation can be considered prognostic factors in patients with BCL11A overexpressing TNBC. Moreover, BCL11A overexpressing TNBCs were associated with a higher proliferation index (> 35%). Among TNBC histotypes, the medullary type of pattern is often associated with variable immunohistochemical expression of basal markers (Rakha et al. 2019). Our previous findings confirmed that medullary and metaplastic carcinomas exhibit higher grades (G3) and higher proliferation index (Ki67 > 30%), while LVI was detected in only 7.4% of medullary carcinomas. Metaplastic carcinoma had poor 5 and 10 year survival in comparison with other histologic types (Sanges et al. 2020).

We found a negative relationship between LVI and BCL11A expression, in contrast with previous results that gave no significant differences (Shen et al. 2017). However, Ugras et al. demonstrated that LVI and nodal metastases were less frequent in TNBC vs other BC subtypes (Ugras et al. 2014). Based on previous findings we could speculate that in BCL11A overexpressing TNBC the worse prognosis is not related to LVI rate.

Our data showed an inverse association between BCL11A overexpression and AR expression levels in TNBCs. Considering that patients with LAR TNBC showed the best OS compared to the other TNBCs subtypes (Masuda et al. 2013), our results might suggest that BCL11A can be a biomarker for more aggressive non luminal TNBCs subgroups. Choi et al. findings could support previous hypothesis, showing that the inhibition of BCL11A and HDAC1/2 effectively reprogramming basal like cancer cells into luminal *A* cells, increasing ER expression and leading to tamoxifen sensitivity (Choi et al.^2022^). In contrast with our results, Wang et al. identified a positive correlation between AR and BCL11A expression by analyzing all BC molecular subtypes (Wang et al. 2020).

Our survival analysis did not show any relationship between BCL11A gene and/or protein expression and patient outcomes. Khaled et al. demonstrated that patients with copy number (CN) gains of BCL11A had a higher rate of relapse and metastasis and a lower rate of survival (Khaled et al. 2015). The differences could be related to the selection of TNBC with basal like phenotype included in the Khaled’s study, compared to our study in which all TNBC phenotypes, included LAR, were all considered.

No nucleotide variants were found in BCL11A exon 4. The literature data demonstrates the presence of different genomic alterations for this gene in malignant diseases, as well as CV amplification, epigenetic deregulation, translocation or abnormal activation upon viral integration (Boelens et al. 2009; Jiang et al. 2013; Yin et al. 2019).

We recognize that our study does have some limitations mainly related to its retrospective nature: key clinical follow-up data were unfortunately not found in medical records.

## Conclusions

Our study highlights the role of BCL11A and its correlation with clinicopathological features of TNBC. BCL11A expression seems to be a poor prognostic factor in TNBC patients. BCL11A may become a prognostic factor for more aggressive non luminal TNBCs subgroups, with the worse prognosis of BCL11A overexpressing TNBC not related to LVI. Furthermore, BCL11A was overexpressed in more aggressive histologic types, such as metaplastic and medullary carcinomas. These results may provide a new paradigm for TNBC classification and a better treatment strategy.

## Supplementary Information

Below is the link to the electronic supplementary material.

Supplementary file1 (DOCX 18 KB)

## Data Availability

The data can be obtained upon a reasonable request from the corresponding author.

## Abbreviations

TNBC: Triple negative breast cancer
BC: Breast cancer
ER: Estrogen receptor
PR: Progesterone receptor
LAR: Luminal androgen receptor
FFPE: Formalin-fixed paraffin-embedded
IHC: Immunohistochemistry
SISH: In situ hybridization
NBT: Normal breast tissue
TMAs: Tissue microarrays
ASCO/CAP: American Society of Clinical Oncology/College of American Pathologists
RIN: RNA integrity number
qRT-PCR: Quantitative real-time PCR
FC: Fold change
TIL: Tumor infiltrating lymphocyte
LIV: Lymph-vascular invasion
OS: Overall survival
CN: Copy number
