# Supplementary material for: Deciphering clinical significance of BCL11A isoforms and protein expression roles in triple-negative breast cancer subtype
Source: J Cancer Res Clin Oncol. 2022 Aug 28;149(7):3951–63. doi: 10.1007/s00432-022-04301-w (PMC10314865; doi:10.1007/s00432-022-04301-w)
Supplement: Supplementary file 1 — Supplementary file1 (DOCX 18 KB) [file 432_2022_4301_MOESM1_ESM.docx]

Deciphering clinical significance of BCL11A isoforms and protein expression roles in Triple-Negative Breast Cancer subtypes

Journal of Cancer Research and Clinical Oncology

Andrea Angius, Giovanna Pira, Paolo Cossu-Rocca, Giovanni Sotgiu, Laura Saderi, Maria Rosaria Muroni, Patrizia Virdis, Daniela Piras, Rallo Vincenzo, Ciriaco Carru, Donatella Coradduzza, Maria Gabriela Uras, Pierina Cottu, Alessandro Fancellu, Sandra Orrù, Paolo Uva, Maria Rosaria De Miglio

Corresponding Authors

Maria Rosaria De Miglio Department of Medicine, Surgery and Pharmacy, University of Sassari, Italy.

E-mail: demiglio@uniss.it

Andrea Angius Institute of Genetic and Biomedical Research (IRGB), CNR, Italy.

E-mail: andrea.angius@irgb.cnr.it

**Table S1. Clinico-pathological features of 343 patients with Triple Negative breast cancer.**

| **Variables** | | **N (%)** |
| --- | --- | --- |
| *Median (IQR) age, years* | | 56 (26–94) |
| *Age, n (%)* | *≤50 years* | 121 (35.4) |
|  | *>50 years* | 221 (64.6) |
| *Site, n (%)* | *Right* | 133 (46.2) |
|  | *Left* | 154 (53.5) |
|  | *Bilateral* | 1 (0.3) |
| *Histologic subtype, n (%)* | *Invasive ductal carcinoma (NST)* | 230 (75.4) |
|  | *Apocrine carcinoma* | 21 (6.8) |
|  | *Medullary carcinoma* | 20 (6.5) |
|  | *Invasive lobular carcinoma* | 15 (4.9) |
|  | *Metaplastic + squamous carcinoma* | 9 (2.9) |
|  | *Papillary carcinoma* | 5 (1.6) |
|  | *Other* | 9 (2.9) |
| *Tumour size, n (%)* | *≤20 mm* | 104 (44.6) |
|  | *>20 mm* | 129 (55.4) |
| *Pathologic tumor classification, n (%)* | *pT1* | 113 (35.0) |
|  | *pT2* | 164 (50.8) |
|  | *pT3* | 25 (7.7) |
|  | *pT4* | 21 (6.5) |
| *Regional lymph nodes involvement, n (%)* | *pN0* | 179 (56.7) |
|  | *pN1* | 81 (25.9) |
|  | *pN2* | 31 (9.9) |
|  | *pN3* | 25 (7.9) |
| *Histologic grade, n (%)* | *G1* | 4 (1.3) |
|  | *G2* | 45 (13.7) |
|  | *G3* | 279 (85.0) |
| *Lymph node ratio* | *≤20* | 248 (79.7) |
|  | *0.21-0.65* | 41 (13.2) |
|  | *>65* | 22 (7.1) |
| *Tumour stage, n (%)* | *1* | 71 (25.7) |
|  | *2* | 141 (51.1) |
|  | *3* | 58 (21.0) |
|  | *4* | 6 (2.2) |
| *Necrosis, n (%)* | *Present* | 150 (61.2) |
|  | *Absent* | 95 (38.8) |
| *Tumor infiltrating lymphocytes, n (%)* | *Present* | 170 (70.5) |
|  | *Absent* | 71 (29.5) |
| *Lymph vascular invasion, n (%)* | *Present* | 98 (41.9) |
|  | *Absent* | 136 (58.1) |
| *Ki67, n (%)* | *≤14%* | 18 (5.4) |
|  | *15-35%* | 75 (22.5) |
|  | *>36* | 240 (72.1) |
| *AR, n (%)* | *<10%* | 140 (78.7) |
|  | *≥10%* | 38 (21.3) |
| *Death, n (%)* | | 124 (36.7) |
| *Protein expression BCL11A* | *Low: 0-1* | 264 (77.0) |
|  | *High: 2-8* | 79 (23.0) |

IQR Interquartile range, N Number
